# Comparison of anti-human T cell globulins on immune reconstitution and early infections after autologous transplant in patients with multiple sclerosis

**DOI:** 10.1038/s41409-025-02730-y

**Published:** 2025-11-27

**Authors:** Johanna Richter, Nico Gagelmann, Felix Fischbach, Kristin Rathje, Lena Kristina Pfeffer, Boris Fehse, Anita Badbaran, Susanna Carolina Berger, Rolf Krause, Evgeny Klyuchnikov, Christine Wolschke, Catherina Lueck, Francis Ayuk, Manuel A. Friese, Christoph Heesen, Nicolaus Kröger

**Affiliations:** 1 Department for Stem Cell Transplantation, University Medical Center Hamburg-Eppendorf, Hamburg, Germany; 2 Institute of Neuroimmunology and Multiple Sclerosis and Department of Neurology, University Medical Center Hamburg-Eppendorf, Hamburg, Germany

**Keywords:** Autotransplantation, Neurological disorders

## Abstract

Autologous hematopoietic stem cell transplantation is an effective therapeutic option for patients with treatment-refractory multiple sclerosis (MS) and may be considered as first line treatment in aggressive forms. Currently, a variety of conditioning and serotherapy regimens are employed across transplant centers. In this study, we compared immune reconstitution at days 30 and 100 post-transplant in MS patients undergoing AHSCT with cyclophosphamide-based conditioning, combined with in vivo T-cell depletion using either polyclonal rabbit anti-thymocyte globulin (ATG; Thymoglobulin, Genzyme-Sanofi) or rabbit anti-T-lymphocyte globulin (ATLG; Grafalon, Neovii). We observed a significantly faster immune reconstitution for CD3^+^, CD3^+^HLA-DR^+^, CD3^+^CD4^+^, CD4^+^CD45RA^+^, CD4^+^CD45RO^+^, CD3^+^CD8^+^, CD8^+^CD45RA^+^, CD8^+^CD45RO^+^, and CD4^+^CD25^+^CD127low cells in patients receiving ATLG compared to ATG at day 30 post-transplant. Although infections resulting in rehospitalization by day 180 were similarly distributed between groups, viral reactivations occurred exclusively in patients receiving ATG. No sign of high grade infectious complications or death was noted.

## Introduction

Multiple sclerosis (MS) is an autoimmune disease of the central nervous system characterized by neuroinflammation and neurodegeneration [1]. Autologous hematopoietic stem cell transplantation (AHSCT) is recommended as a clinically proven therapeutic option for selected patients with treatment-resistant neurological autoimmune diseases and may also be considered as an upfront option in aggressive disease [2–5]. Studies indicate that AHSCT offers superior outcomes in reducing relapses, achieving NEDA and limiting MRI activity compared to standard disease-modifying therapies [6–8]. High-dose chemotherapy with lymphodepleting serotherapy followed by AHSCT aims to eliminate the aberrant immune system and restore one that is more self-tolerant, supporting sustained remission [9]. Although various chemotherapy regimens are available, the EBMT guidelines recommend cyclophosphamide with anti-thymocyte globulin (Cy-ATG) or BEAM combined with ATG (BEAM-ATG) as regimen for MS patients [3]. Serotherapy with polyclonal ATG consists of purified IgG from rabbits, horses, or occasionally goats immunized with human thymocytes or T cell lines. Among available formulations, anti-thymocyte globulin (ATG; Thymoglobulin, Genzyme-Sanofi, Cambridge, Massachusetts, USA) is derived from rabbits immunized with human thymocytes, while anti-T-lymphocyte globulin (ATLG; Grafalon®, Neovii, Rapperswil, Switzerland) is generated by immunizing rabbits with the Jurkat T-cell line resembling activated T cells [10]. To date, the selection of anti-T cell serotherapy is predominantly determined by therapeutic availability, institutional protocols, and/or participation in clinical trials. Real-world data from EBMT centers showed that although all centers employed polyclonal anti-thymocyte globulin, there was substantial variation in both the total dose and administration schedule of serotherapy [11]. Thus, comparative data on serotherapy approaches are needed to eventually support the development of a harmonized lymphodepleting regimen for patients undergoing AHSCT for MS.

This study aims to evaluate the effects of ATG and ATLG on immune reconstitution and early infectious complications following cyclophosphamide-based AHSCT in patients with MS.

## Methods

### Data source

The study included 63 patients with relapsed-remitting MS (RRMS; *n* = 34), primary-progressive MS (PPMS; *n* = 20) or secondary-progressive MS (SPMS; *n* = 9) who underwent first AHSCT at the Department of Stem Cell Transplantation at the University Medical Center Hamburg-Eppendorf, Germany, between 2020 and 2025. The study was approved by the institutional review board and was conducted in accordance with the Declaration of Helsinki and Good Clinical Practice guidelines. All patients provided written informed consent approved by the Ethic committee of the chamber of physicians Hamburg (2022-100940-BO-ff).

### Patient characteristics and conditioning regimen

**Fig. 1:**
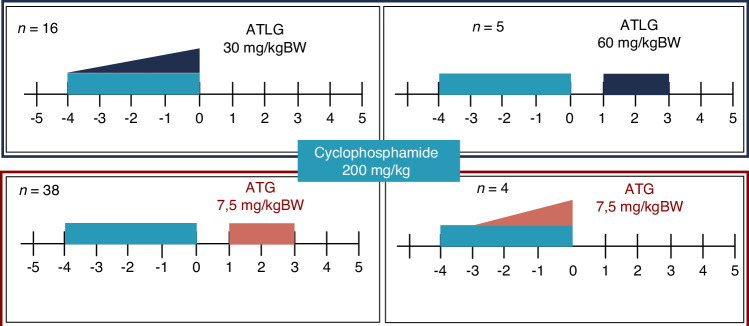
Different conditioning regimen. Overview of conditioning regimens including administration schedule and dosing of ATG and ATLG. The x-axis depicts the timeline of the conditioning regimen, showing the sequence and days of administration of each component relative to transplantation (day 0). ATG anti-thymocyte globulin, ATLG anti-T-lymphocyte globulin, BW body weight.

All patients received conditioning chemotherapy consisting of four doses of cyclophosphamide at 50 mg/kg body weight, administered from day −5 to day −1. A total of 42 patients received ATG, while 21 patients were treated with ATLG (see Fig. 1). In the ATG cohort, 38 patients (post-ATG group) received a cumulative dose of 7.5 mg/kg body weight on days +1 and +2 post-transplant, while 4 patients (pre-ATG group) received the same total dose in a fractionated schedule from day −3 to day −1 prior to transplantation. In the ATLG group, 16 patients (pre-ATLG group) received a total dose of 30 mg/kg body weight in escalating doses from day −4 to day −1, and 5 patients (post-ATLG group) received a cumulative dose of 60 mg/kg body weight on days +1 and +2 post-transplant.

### Immune phenotyping

Peripheral blood lymphocyte immunophenotyping was conducted via flow cytometry to characterize T cells (CD3^+^), activated T cells (CD3^+^/HLA-DR^+^), T helper (CD3^+^/CD4^+^), and cytotoxic T cells (CD3^+^/CD8^+^), B cells (CD19^+^), regulatory B cells (CD19^+^/CD5^+^/CD1d^+^), memory (CD19^+^/CD27^+^) and naïve B cells (CD19^+^/CD27^−^/CD10^+^), natural killer (CD56⁺/CD3⁻) and natural killer T cells (CD56⁺/CD3⁺), naïve (CD4^+^/CD45RA^+^) and memory T helper cells (CD4^+^/CD45RO^+^), naïve (CD8^+^/CD45RA^+^) and memory cytotoxic T cells (CD8^+^/CD45RO^+^), γδ T cells (TCRγδ^+^/CD3^+^), and regulatory T cells (CD4^+^/CD25^+^/CD127low). Immune status was systematically assessed on days +30 and +100 post-AHSCT to evaluate immune reconstitution across treatment groups.

### Viral reactivations

Epstein-Barr virus (EBV) DNA load was assessed using PCR performed on whole blood samples as previously described [12]. Significant EBV DNA detection was defined as ≥1000 copies per microliter.

For cytomegalovirus (CMV) quantification, real-time quantitative (q)PCR was carried out on genomic DNA isolated from whole blood samples using QIAamp DNA Blood Minikit (Qiagen). QPCR was performed in duplex reactions to simultaneously measure both the number of CMV copies and the number of cells using a diploid reference gene (hematopoietic cell kinase, HCK) as target as described [13]. For absolute and relative quantification, ct and delta-ct values (ctCMV–ctHCK) were used. CMV reactivation was defined as high viral load corresponding to a delta-ct ≤12 and/or >1000 viral copies per ml.

### Statistical analysis

Primary endpoint of this study was to compare immune reconstitution between the ATG and the ATLG group at the two time points. Secondary endpoints included infections leading to rehospitalization until day 180, infections leading to intensive care treatment or death, EBV and cytomegalovirus reactivations.

Patient characteristics were described with median and range for continuous variables and frequencies for categorical variables. Immune profiles and dynamics were compared in a categorical and time-dependent fashion using Wilcoxon tests. All analyses were performed using R version 4.4.2 (R Foundation for Statistical Computing, Vienna, Austria).

## Results

### Patients

Baseline characteristics were comparably distributed when analysis was restricted to the main treatment groups, specifically patients receiving post-transplant ATG (*n* = 38) and those receiving pre-transplant ATLG (*n* = 16) (see Table 1). However, patients receiving pre-transplant ATLG had a shorter follow-up period (*p* = 0.03). Both groups exhibited similar hematopoietic recovery, with median neutrophil and platelet engraftment occurring at 10 days.

**Table 1:** Patient characteristics.

Characteristic	Thymo	ATLG	*P*
Age, median (*r*)	33 (22–48)	37 (25–49)	
Sex, *n* (%)			0.22
Male	20 (53)	11 (69)	
Female	18 (48)	5 (31)	
ECOG, *n* (%)			0.72
0	5 (14)	3 (23)	
1	26 (70)	8 (62)	
2	6 (16)	2 (15)	
Unknown	1	3	
Multiple sclerosis form, *n* (%)			0.40
PPMS	12 (32)	8 (50)	
RRMS	21 (55)	7 (44)	
SSMP	5 (13)	1 (6)	
Conditioning, *n* (%)			
Cy	38 (100)	16 (100)	
Time from diagnosis to auto in years, median (range)	5.7 (1.3–21.8)	7.0 (2.1–11.5)	0.61
Follow-up in years, months (range)	9.1 (0.4–27)	3.1 (0.5–6.7)	0.03
Leukocyte engraftment, median (*r*)	10 (7–12)	10 (7–12)	0.91
Platelet engraftment, median (*r*)	10 (5–14)	10 (4–13)	0.60

### Immune reconstitution day 30

**Fig. 2:**
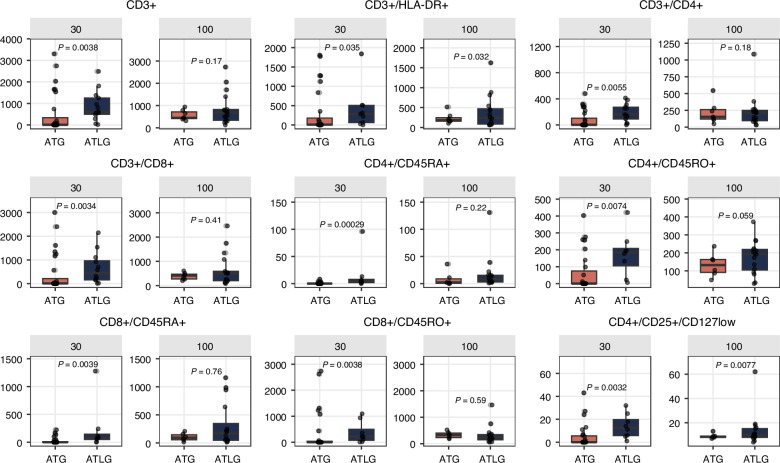
T cell reconstitution at day 30 and day 100 comparing pretransplant ATLG to posttransplant ATG. Counts of T cell subpopulations at day 30 and day 100 comparing pre-transplant ATLG with post-transplant ATG. ATG anti-thymocyte globulin, ATLG anti-Tlymphocyte globulin.

**Fig. 3:**
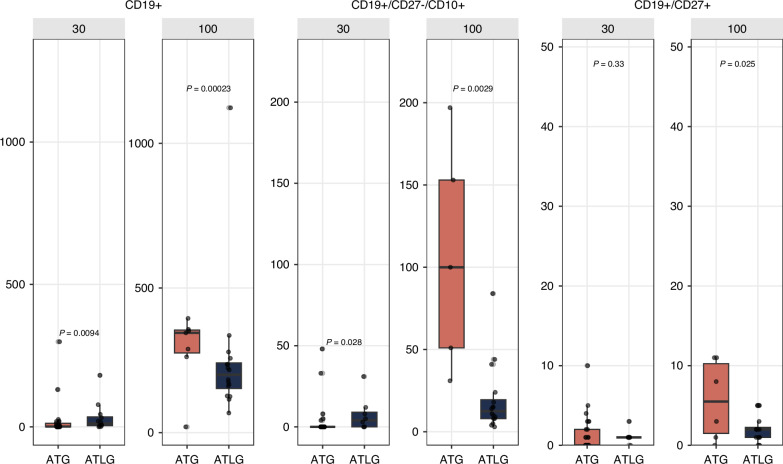
B cell reconstitution at day 30 and day 100 comparing pretransplant ATLG to posttransplant ATG. Counts of B cell subpopulations at day 30 and day 100 comparing pre-transplant ATLG with post-transplant ATG. ATG anti-thymocyte globulin, ATLG anti-Tlymphocyte globulin.

Comparing the main treatment groups, we observed a significantly faster immune reconstitution at day 30 in patients receiving pre-transplant ATLG compared to those receiving post-transplant ATG as indicated by higher counts of CD3+, CD3^+^/CD4^+^, CD4^+^/CD45RO^+^, CD3^+^/CD8^+^ T-cell subsets (see Fig. 2). Additionally, significantly higher counts of regulatory T cells (CD4+/CD25+/CD127low) and B cells (CD19+), especially naive B cells (CD19^+^/CD27^−^/CD10^+^), were observed in the pre-ATLG group. (Fig. 3)

**Fig. 4:**
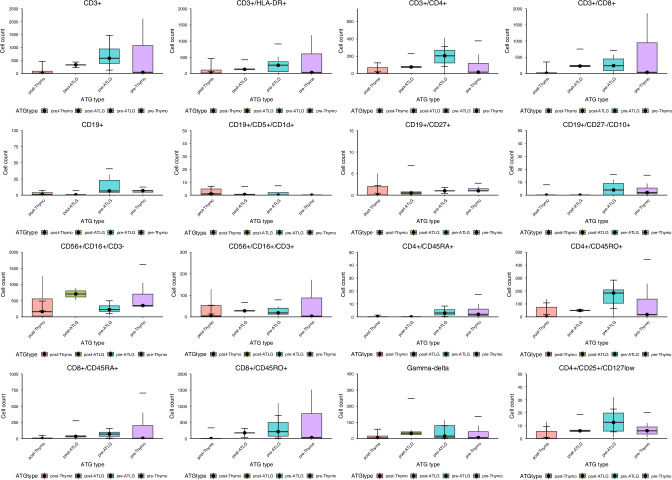
Immune reconstitution at day 30 comparing pretransplant and posttransplant administration of ATLG and ATG. Comparison of early immune subset recovery at day 30 between pre- and post-transplant ATLG and ATG. ATG antithymocyte globulin, ATLG anti-T-lymphocyte globulin.

Beyond the primary cohorts treated with either post-transplant ATG or pre-transplant ATLG, we additionally included subgroups of patients who received either pre-transplant ATG or post-transplant ATLG. Although the limited number of cases precluded meaningful statistical comparison, we continued to observe higher counts for CD3+, CD3^+^/CD4^+^, CD4^+^/CD45RO^+^, CD3^+^/CD8^+^ and CD4+/CD25+/CD127low T cell subsets in the post-transplant ATLG group compared to the pre- and post-transplant ATG groups at day 30 (see Fig. 4). Patients who received pre-transplant serotherapy exhibited comparable B cell counts across groups, with levels exceeding those observed in recipients of post-transplant serotherapy.

### Immune reconstitution day 100

At day 100 post-transplant, T-cell reconstitution was not significantly more advanced in recipients of pre-transplant ATLG compared to those who received post-transplant ATG. We observed significantly higher counts for CD19+, CD19+/CD27−/CD10+, and CD19+/CD27+B cell subsets in the post-transplant ATG group compared to the pre-transplant ATLG group.

**Fig. 5:**
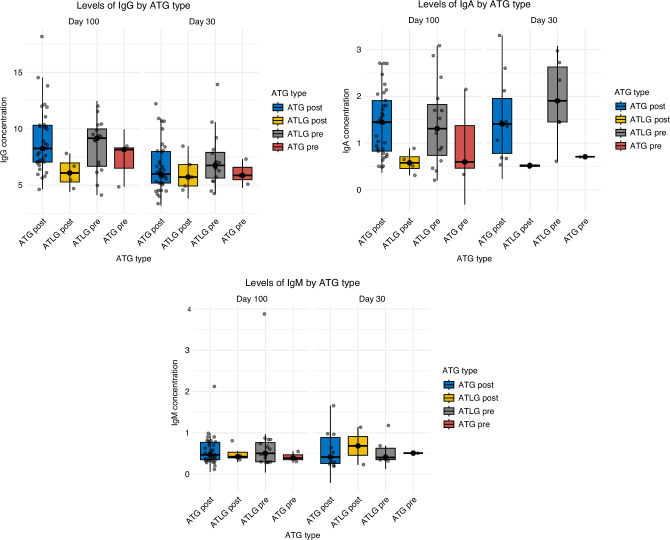
Immunoglobulin measurements. Immunoglobulin measurements over time in patients receiving pre- and post-transplant ATLG or ATG. ATG antithymocyte globulin, ATLG anti-T-lymphocyte globulin.

Regarding immunoglobulin measurements, no significant differences were observed between the groups at day 30 or day 100 (see Fig. 5).

### Complications

No mortality or need for intensive care support was observed in any patient. Within the first 180 days post-transplant, eight infection-related rehospitalizations were recorded, with a mean hospital stay of 7.3 (2–18) days. The majority of infections were categorized as fever of unknown origin (*n* = 5), with single cases of CMV reactivation, pulmonary infection, and sinusitis also documented. Infection-related rehospitalizations were similarly distributed in the ATG group (15.8%, *n* = 6) and the ATLG group (12.5%, *n* = 2). Viral reactivations were observed exclusively in the ATG cohort. CMV reactivation was detected in five patients, four of whom were managed in the outpatient setting with oral Valaciclovir. EBV-DNA was detected in the peripheral blood of one patient, which resolved spontaneously without the need for therapeutic intervention.

## Discussion

This study provides valuable insights into the differential effects of ATG and ATLG on immune reconstitution in MS patients undergoing AHSCT. ATLG was associated with significantly faster T cell reconstitution at day 30 post-AHSCT compared to ATG. This effect seems to occur irrespective of the timing of serotherapy administration. While some studies comparing the two ATG formulations in the context of allogeneic stem cell transplantation observed no differences within immune reconstitution [14, 15], others emphasize the here-described delayed reconstitution within the T-cell compartment in patients receiving ATG [16–18]. This effect may partly result from the more pronounced T-cell depletion mediated by ATG. Due to differences in the sources of immunization cell, ATG formulations exhibit distinct antigen specificities: ATG displays broad reactivity, targeting a wide range of surface antigens, including T-cell markers (CD2, CD3, CD4, CD6, and CD8), as well as molecules expressed on B cells, natural killer cells, macrophages, dendritic cells, and major histocompatibility complex proteins (HLA class I and HLA-DR). In contrast, ATLG has a more limited antigen profile and lacks significant reactivity against CD3, CD4, and HLA-DR, resulting in a comparatively reduced immunosuppressive effect [19]. Besides differences in antigen specificity, ATG and ATLG also vary in the pharmacokinetics of their active compounds: a pediatric study on allogeneic transplantation demonstrated faster clearance of active ATLG compared to ATG [16]. The combination of a narrower antigen profile and reduced active drug persistence may explain the more rapid T-cell reconstitution observed with ATLG.

Conversely, the greater cytotoxicity of ATG toward human thymocytes may impair thymic regeneration [20]. As reactivation of thymopoiesis plays a pivotal role in immune reconstitution after AHSCT in MS patients [21], thymic cytotoxicity might also contribute to delayed T-cell recovery in patients receiving ATG.

In line with the accelerated immune reconstitution of T cell subsets, patients receiving ATLG showed significantly higher counts of regulatory T cells than those treated with ATG. Following AHSCT, MS patients experience a transient expansion of regulatory T cells during the early post-transplant phase. Although their levels typically return to baseline over time, this initial surge may play a pivotal role in re-establishing immune tolerance by exerting enhanced regulatory activity during a critical phase for the naïve, newly regenerated immune system [22]. In a study comparing patients with BEAM conditioning for Non-Hodgkin Lymphoma (NHL) and MS, where only the MS cohort received ATG, Treg cell frequencies in MS patients showed a significant and lasting increase after transplantation, while Treg levels remained stable in NHL patients. This effect may reflect a correction of immune dysregulation that contributes to the containment of neuroinflammation after AHSCT, or may alternatively be attributable to the immunomodulatory effects of ATG [23]. Our data suggests a more pronounced Treg expansion following ATLG. Further studies are warranted to investigate whether the observed effects translate into differences in clinical outcomes.

Regarding B cell reconstitution, we observed earlier reconstitution in the pretransplant ATLG group compared to the posttransplant ATG group. Rather than a substance-specific effect, this phenomenon seems to be more driven by the timing of serotherapy administration, as similar high counts were observed in patients receiving pre-transplant ATG. Our findings emphasize that, in addition to the serotherapy used, the timing of application might influence post-transplant immune reconstitution. In contrast, we observed a faster reconstitution of B cells within patients receiving ATG compared to ATLG on day 100. Similar dynamics were observed comparing high-dose ATG and ATLG in the setting of allogeneic transplantation [16]. The reason for this remains unclear.

In addition to administration timing, dosing differences may influence variations in immune reconstitution between ATG- and ATLG-treated patients. In ASCT for MS, most centers use a ATG dose of 6 mg/kg BW [11], which is slightly lower than the dose used in this study. While dose-finding studies did not identify significant differences between 6 and 8 mg/kg BW in the allogenic setting [24], recent data of ASCT in MS patients indicate significantly better progression-free survival in patients receiving ≤6 mg/kg BW [25]. For ATLG, applied doses ranged from 7.5 to 90 mg/kg BW with no single dose consistently favored across centers [11]. Studies in allogenic transplantation have demonstrated dose-dependent differences in immune reconstitution for both ATG [26] and ATLG [27], yet a clear dosing equivalence still needs to be defined [28, 29].

While infections leading to rehospitalization were nearly equally distributed among patients receiving ATG and those receiving ATLG, virus reactivations were solely observed in the ATG group. These results could be explained by the observed slower T cell reconstitution within the ATG group, thereby emphasizing that delayed T cell reconstitution renders patients at risk for infectious complications, in particular virus reactivations. Studies comparing ATG and ATLG in allogeneic stem cell [28, 30, 31] and kidney transplantation [32–34] support these findings by also reporting a higher number of viral infections in patients receiving ATG compared to those receiving ATLG.

We acknowledge certain limitations in the presented study. The limited sample size and retrospective design of the study increase its susceptibility to bias. Also, due to the small sample size of patients receiving posttransplant ATLG and pretransplant ATG, we were unable to include these groups in the comparable statistical analysis. The limited follow-up period enabled only the analysis of early immune reconstitution and infectious complications. Because reconstitution of the adaptive immune is reported to take up to 2 years post-AHSCT [22], long-term assessment could provide a more complete picture of differences with regard to immune reconstitution trends and infectious complications in patients receiving ATG or ATLG. We aim to extend and deepen the analysis of the provided data in future work.

To the best of our knowledge, this is the first study comparing immune reconstitution in MS patients following AHSCT with either ATG or ATLG. Patients receiving pretransplant ATLG demonstrated a significantly faster immune reconstitution in T cell subsets and fewer early infectious complications than patients receiving post-transplant ATG. Our study thereby not only contributes to the understanding of the different immunogenic effects of these two serotherapies but also provides important insights regarding the safety of MS patients undergoing AHSCT. Further prospective studies are warranted to evaluate the potential impact of different serotherapy agents on clinical outcomes.

## Data Availability

Questions regarding data sharing should be addressed to the corresponding author.

## References

[1] Woo MS, Engler JB, Friese MA. The neuropathobiology of multiple sclerosis. Nat Rev Neurosci. 2024;25:493–513. 38789516 10.1038/s41583-024-00823-z

[2] Alexander T, Greco R. Hematopoietic stem cell transplantation and cellular therapies for autoimmune diseases: overview and future considerations from the Autoimmune Diseases Working Party (ADWP) of the European Society for Blood and Marrow Transplantation (EBMT). Bone Marrow Transpl. 2022;57:1055–62. 10.1038/s41409-022-01702-w PMC9109750 35578014

[3] Sharrack B, Saccardi R, Alexander T, Badoglio M, Burman J, Farge D, et al. Autologous haematopoietic stem cell transplantation and other cellular therapy in multiple sclerosis and immune-mediated neurological diseases: updated guidelines and recommendations from the EBMT Autoimmune Diseases Working Party (ADWP) and the Joint Accreditation Committee of EBMT and ISCT (JACIE). Bone Marrow Transpl. 2019;55:283. 10.1038/s41409-019-0684-0 PMC6995781 31558790

[4] Snowden JA, Saccardi R, Allez M, Ardizzone S, Arnold R, Cervera R, et al. Haematopoietic SCT in severe autoimmune diseases: updated guidelines of the European Group for Blood and Marrow Transplantation. Bone Marrow Transpl. 2011;47:770. 10.1038/bmt.2011.185 PMC3371413 22002489

[5] Muraro PA, Mariottini A, Greco R, Burman J, Iacobaeus E, Inglese M, et al. Autologous haematopoietic stem cell transplantation for treatment of multiple sclerosis and neuromyelitis optica spectrum disorder—recommendations from ECTRIMS and the EBMT. Nat Rev Neurol. 2025;21:140–58. 39814869 10.1038/s41582-024-01050-x

[6] Boffa G, Lapucci C, Sbragia E, Varaldo R, Raiola AM, Currò D, et al. Aggressive multiple sclerosis: a single-centre, real-world treatment experience with autologous haematopoietic stem cell transplantation and alemtuzumab. Eur J Neurol. 2020;27:2047–55. 32418281 10.1111/ene.14324

[7] Häußler V, Ufer F, Pöttgen J, Wolschke C, Friese MA, Kröger N, et al. aHSCT is superior to alemtuzumab in maintaining NEDA and improving cognition in multiple sclerosis. Ann Clin Transl Neurol. 2021;8:1269–78. 33949790 10.1002/acn3.51366 PMC8164852

[8] Braun B, Fischbach F, Richter J, Pfeffer LK, Fay H, Reinhardt S, et al. Benefits of aHSCT over alemtuzumab in patients with multiple sclerosis besides disability and relapses: sustained improvement in cognition and quality of life. Mult Scler Relat Disord. 2024;82. Available from: https://pubmed.ncbi.nlm.nih.gov/38176284/. 10.1016/j.msard.2023.105414 38176284

[9] Abrahamsson S, Muraro PA. Immune re-education following autologous hematopoietic stem cell transplantation. Autoimmunity. 2008;41:577–84. 18958748 10.1080/08916930802197081

[10] Mohty M. Mechanisms of action of antithymocyte globulin: T-cell depletion and beyond. Leukemia. 2007;21:1387–94. 17410187 10.1038/sj.leu.2404683

[11] Ismail A, Nitti R, Sharrack B, Badoglio M, Ambron P, Labopin M, et al. ATG and other serotherapy in conditioning regimens for autologous HSCT in autoimmune diseases: a survey on behalf of the EBMT Autoimmune Diseases Working Party (ADWP). Bone Marrow Transplant. 2024;59. Available from https://pubmed.ncbi.nlm.nih.gov/39143182/. 10.1038/s41409-024-02383-3 PMC11530368 39143182

[12] Gatto F, Cassina G, Broccolo F, Morreale G, Lanino E, Di Marco E, et al. A multiplex calibrated real-time PCR assay for quantitation of DNA of EBV-1 and 2. J Virol Methods. 2011;178:98–105. 21903135 10.1016/j.jviromet.2011.08.022

[13] Fehse B, Chukhlovin A, Kühlcke K, Marinetz O, Vorwig O, Renges H, et al. Real-time quantitative Y chromosome-specific PCR (QYCS-PCR) for monitoring hematopoietic chimerism after sex-mismatched allogeneic stem cell transplantation. J Hematother Stem Cell Res. 2001;10:419–25. 11454317 10.1089/152581601750289028

[14] Notarantonio AB, Morisset S, Piucco R, Pérès M, Boulangé L, Alitcher A, et al. Differential clinical and immunological impacts of anti-T-lymphocyte globulin (ATLG) vs. anti-thymocyte globulin (ATG) in preventing graft-versus-host disease post-allogeneic hematopoietic stem cell transplantation: a comparative study. Am J Hematol. 2025;100:626. 39905816 10.1002/ajh.27619 PMC11886505

[15] Nirmal G, Kharya G, Shankar R, Singh S, Paul S, Choudhary M, et al. A comparative analysis of low dose grafalon® versus thymoglobuline® as serotherapy in hematopoietic stem cell transplant in pediatric and young adult population. Pediatr Hematol Oncol. 2024; Available from https://www.tandfonline.com/doi/abs/10.1080/08880018.2024.2398523. 10.1080/08880018.2024.2398523 39310983

[16] Oostenbrink LVE, Jol-Van Der Zijde CM, Kielsen K, Jansen-Hoogendijk AM, Ifversen M, Müller KG, et al. Differential elimination of anti-thymocyte globulin of Fresenius and genzyme impacts T-cell reconstitution after hematopoietic stem cell transplantation. Front Immunol. 2019;10. Available from https://pubmed.ncbi.nlm.nih.gov/30894854/. 10.3389/fimmu.2019.00315 PMC6414431 30894854

[17] Mensen A, Na IK, Häfer R, Meerbach A, Schlecht M, Pietschmann ML, et al. Comparison of different rabbit ATG preparation effects on early lymphocyte subset recovery after allogeneic HSCT and its association with EBV-mediated PTLD. J Cancer Res Clin Oncol. 2014;140:1971–80. 24962343 10.1007/s00432-014-1742-z PMC11824132

[18] Terasako K, Sato K, Sato M, Kimura SI, Nakasone H, Okuda S, et al. The effect of different ATG preparations on immune recovery after allogeneic hematopoietic stem cell transplantation for severe aplastic anemia. Hematology. 2010;15:165–9. 20557676 10.1179/102453309X12583347113852

[19] Popow I, Leitner J, Grabmeier-Pfistershammer K, Majdic O, Zlabinger GJ, Kundi M, et al. A comprehensive and quantitative analysis of the major specificities in rabbit antithymocyte globulin preparations. Am J Transpl. 2013;13:3103–13. 10.1111/ajt.12514 24168235

[20] Na IK, Wittenbecher F, Dziubianau M, Herholz A, Mensen A, Kunkel D, et al. Rabbit antithymocyte globulin (thymoglobulin) impairs the thymic output of both conventional and regulatory CD4+ T cells after allogeneic hematopoietic stem cell transplantation in adult patients. Haematologica. 2013;98:23–30. 22801968 10.3324/haematol.2012.067611 PMC3533656

[21] Muraro PA, Douek DC, Packer A, Chung K, Guenaga FJ, Cassiani-Ingoni R, et al. Thymic output generates a new and diverse TCR repertoire after autologous stem cell transplantation in multiple sclerosis patients. J Exp Med. 2005;201:805. 15738052 10.1084/jem.20041679 PMC2212822

[22] Mariottini A, Cencioni MT, Muraro PA. Immune cell reconstitution following autologous hematopoietic stem cell transplantation in multiple sclerosis. Handb Clin Neurol. 2024;202:55–74. 39111918 10.1016/B978-0-323-90242-7.00003-1

[23] Moore JJ, Massey JC, Ford CD, Khoo ML, Zaunders JJ, Hendrawan K, et al. Prospective phase II clinical trial of autologous haematopoietic stem cell transplant for treatment refractory multiple sclerosis. J Neurol Neurosurg Psychiatry. 2019;90:514–21. 30538138 10.1136/jnnp-2018-319446

[24] Meijer E, Cornelissen JJ, Löwenberg B, Verdonck LF. Antithymocyteglobulin as prophylaxis of graft failure and graft-versus-host disease in recipients of partially T-cell—depleted grafts from matched unrelated donors: a dose-finding study. Exp Hematol. 2003;31:1026–30. 14585365 10.1016/s0301-472x(03)00204-2

[25] Kazmi M, Muraro PA, Mehra V, Gabriel I, De Matteis E, Brittain G, et al. Autologous haematopoietic stem cell transplantation for multiple sclerosis in the UK: a 20-year retrospective analysis of activity and haematological outcomes from the British Society of Blood and Marrow Transplantation and Cellular Therapy (BSBMTCT). Br J Haematol. 2025. https://pubmed.ncbi.nlm.nih.gov/40500866/. 10.1111/bjh.20199 PMC12436235 40500866

[26] Meijer E, Bloem AC, Dekker AW, Verdonck LF. Effect of antithymocyte globulin on quantitative immune recovery and graft-versus-host disease after partially T-cell-depleted bone marrow transplantation: a comparison between recipients of matched related and matched unrelated donor grafts. Transplantation. 2003;75:1910–3. 12811256 10.1097/01.TP.0000065737.60591.9D

[27] Massoud R, Klyuchnikov E, Gagelmann N, Zabelina T, Wolschke C, Ayuk F, et al. Impact of Anti-T-lymphocyte globulin dosing on GVHD and Immune reconstitution in matched unrelated myeloablative peripheral blood stem cell transplantation. Bone Marrow Transpl. 2022;57:1548–55. 10.1038/s41409-022-01666-x PMC9532245 35831408

[28] Zhang H, Zhou Y, Zhao K, Cui J, Zhang X, Wen R, et al. Comparison of ATG-thymoglobulin with atg-fresenius in patients with hematological malignancies who undergo allogeneic hematopoietic stem cell transplantation: a propensity score-matched analysis. Ann Hematol. 2025;104:1907. 40016396 10.1007/s00277-025-06267-4 PMC12031750

[29] Baron F, Mohty M, Blaise D, Socié G, Labopin M, Esteve J, et al. Anti-thymocyte globulin as graft-versus-host disease prevention in the setting of allogeneic peripheral blood stem cell transplantation: a review from the Acute Leukemia Working Party of the European Society for Blood and Marrow Transplantation. Haematologica. 2017;102:224–34. 27927772 10.3324/haematol.2016.148510 PMC5286931

[30] Zhou L, Gao Zyong, Lu DP. Comparison of ATG-thymoglobulin with ATG-Fresenius for Epstein-Barr virus infections and graft-versus-host-disease in patients with hematological malignances after haploidentical hematopoietic stem cell transplantation: a single-center experience. Ann Hematol. 2020;99:1389. 32291495 10.1007/s00277-020-04014-5 PMC7222941

[31] Falicovich I, Nachmias B, Elias S, Zimran E, Shaulov A, Stepensky P, et al. Low dose ATG-Fresenius for GVHD prophylaxis: a comparative study with ATG-Thymoglobulin. Front Immunol. 2025;16:1526513. 39931058 10.3389/fimmu.2025.1526513 PMC11807999

[32] Song T, Yin S, Li X, Jiang Y, Lin T. Thymoglobulin vs. ATG-Fresenius as induction therapy in kidney transplantation: a Bayesian network meta-analysis of randomized controlled trials. Front Immunol. 2020;11. Available from https://pubmed.ncbi.nlm.nih.gov/32318057/. 10.3389/fimmu.2020.00457 PMC7146975 32318057

[33] Burkhalter F, Schaub S, Bucher C, Gürke L, Bachmann A, Hopfer H, et al. A comparison of two types of rabbit antithymocyte globulin induction therapy in immunological high-risk kidney recipients: a prospective randomized control study. PLoS ONE. 2016;11:e0165233. 27855166 10.1371/journal.pone.0165233 PMC5113896

[34] Ducloux D, Kazory A, Challier B, Coutet J, Bresson-Vautrin C, Motte G, et al. Long-term toxicity of antithymocyte globulin induction may vary with choice of agent: a single-center retrospective study. Transplantation. 2004;77:1029–33. 15087766 10.1097/01.tp.0000116442.81259.60

